# Direct transfer of electron microscopy samples to wetted carbon and graphene films via a support floatation block

**DOI:** 10.1016/j.jsb.2020.107677

**Published:** 2021-03

**Authors:** Natàlia de Martín Garrido, Wencheng Fu, Kailash Ramlaul, Zining Zhu, David Miller, Daniel Boehringer, Christopher H.S. Aylett

**Affiliations:** a Section for Structural and Synthetic Biology, Department of Infectious Disease, Imperial College London, London, United Kingdom; b State Key Laboratory of Microbial Metabolism, School of Life Sciences & Biotechnology, Shanghai Jiao Tong University, Shanghai, China; c Imperial College Advanced Hackspace, Imperial College London, London, United Kingdom; d Cryo-EM Knowledge Hub (CEMK), ETH Zurich, Zurich, Switzerland

**Keywords:** Carbon, Graphene, 3D-printing, Sample support, Floatation, Wetted transfer

## Abstract

Support films are commonly used during cryo-EM specimen preparation to both immobilise the sample and minimise the exposure of particles at the air-water interface. Here we report preparation protocols for carbon and graphene supported single particle electron microscopy samples using a novel 3D-printed sample transfer block to facilitate the direct, wetted, movement of both carbon and graphene supports from the substrate on which they were generated to small volumes (10 μL) of sample. These approaches are simple and inexpensive to implement, minimise hydrophobic contamination of the support films, and are widely applicable to single particle studies. Our approach also allows the direct exchange of the sample buffer on the support film in cases in which it is unsuitable for vitrification, e.g. for samples from centrifugal density gradients that help to preserve sample integrity.

## Highlights

• Design of a sample-support transfer block for negative stain and cryo-EM.
• Direct wetted transfer of 10 μL samples to carbon.
• Direct wetted transfer of 10 μL samples to graphene.
• Buffer exchange from 10 μL sample volumes *in situ* within the block.

## Graphical abstract

**Figure:**
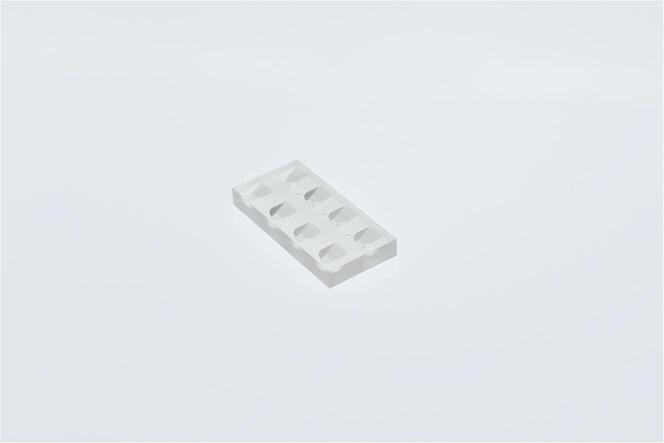

## 1 Introduction

One of the main challenges in *cryogenic electron microscopy* (cryo-EM) is to prepare specimens in such a way that the molecular structures under study remain well preserved in the vacuum of the microscope for *single-particle analysis* (SPA). To achieve this, purified suspensions of the macromolecular complexes in question are plunge-frozen in liquid ethane, preventing the crystallisation of water during the freezing process, and resulting in vitrified complexes with their native structures preserved (McDowall et al., 1983, Adrian et al., 1984, Dubochet et al., 1988).

Samples for transmission EM must be thin in order to preserve phase contrast and prevent multiple scattering events within the sample (Leapman and Ornberg, 1988, Grimm et al., 1996), both of which result in uninterpretable images. This requirement for a very limited ice thickness increases the possibility of particle adsorption at the air-water interface (Noble et al., 2018a). The interaction of macromolecules with an air-water interface results in a wide range of detrimental effects, including denaturation, dissociation, and preferential orientation, thereby reducing the number of suitable particles for analysis and limiting the completeness of reconstructions during image processing and structure determination (Naydenova and Russo, 2017, Glaeser, 2018, D’Imprima et al., 2019). Additionally, when vitrified specimens are irradiated with the electron beam, they move within the field of view and build-up charge (Henderson and Glaeser, 1985, Brink et al., 1998, Berriman and Rosenthal, 2012), which degrades the final image, and limits the resolution obtained. This movement can be affected by the sample support itself (Russo and Passmore, 2014b), and therefore support choice is a key variable during specimen preparation.

The current standard support for specimen preparation is a perforated amorphous carbon foil, with 1–2 μm diameter holes placed across a 3 mm metal mesh grid (Russo and Passmore, 2016). During vitrification, the sample is deposited across the holes, which are typically located in regular arrays. An even distribution of the holes facilitates the imaging process through the use of automated data acquisition software which capture images within the holes. The most popular metal for the grid is copper, although other materials such as gold can be used. Samples can be either deposited on the grid to form a thin layer of liquid spanning through the holes, or onto an additional thin layer of another material, which provides an adherent surface and minimises contact with the air-water interface before vitrification (Russo and Passmore, 2016). Carbon is relatively electron-transparent, and thin and flat carbon films can be easily prepared by deposition onto freshly cut mica using a carbon evaporator at high vacuum (Fujiyoshi, 1998). However, standard perforated amorphous carbon foils also have limitations, the most relevant being movement during electron irradiation (Brilot et al., 2012), and contribution to specimen charging (Russo and Passmore, 2014b). Ideally, after vitrification macromolecules retain the particle distribution, orientation and structure that they had in solution. However, many macromolecules of interest tend to be problematic to prepare as vitrified samples, with the main issues being adsorption to the air-water interface, preferential orientation, and observation of decreased particle number in comparison to that expected given the concentration before vitrification (Glaeser, 2018). Several different approaches to overcome these issues are commonly used, often in parallel: stabilisation of the sample in solution through either buffer modification or cross-linking, using a detergent to cover the air-water interface, using a rapid plunge-freezing robot (Noble et al., 2018b), and immobilisation of the sample through the use of an additional support film.

SPA requires an homogeneous and stable sample for the determination of the three-dimensional structure through the computational averaging of images of identical or similar conformations of the molecule under investigation (Frank et al., 1978). Optimisation of the buffer conditions to stabilise the sample in solution is therefore usually an initial step in cryo-EM. Most popular strategies consist of the addition of stabilising co-solutes, such as polyamines, glycerol, or trehalose, and the optimisation of the pH and ionic strength for the macromolecule in question. However, some of these buffer additives can be incompatible with vitrification. Sample stabilisation requires further optimisation when working with complexes of multiple macromolecules. In fact, most proteins regulating important cellular functions form weak and dynamic multiprotein complexes that can oscillate between various conformational states (Alberts, 1998), thus reducing their stability in solution. Each complex needs specific conditions to remain stable in an aqueous environment; if the *dissociation constant* (K_d_) is known, one can estimate the concentration where the complex will remain intact. If this concentration is prohibitively high, cross-linking agents must be utilised to hold subunits together after dilution. Although the addition of a cross-linking agent may be very beneficial to stabilise the complex in solution, it can also introduce artefacts created by non-specific interactions, inter-particle fixation and aggregation. Density gradient crosslinking, which was first investigated by Kastner and colleagues as “GraFix” (Kastner et al., 2008, Herzog et al., 2009, Golas et al., 2009, Stark, 2010), is a popular means by which such artefacts may be avoided as multiprotein complexes are exposed to a mild concentration of cross-linking agent during sedimentation by centrifugation through a density gradient. Normally, density gradients successfully stabilise dynamic protein complexes, however they usually require subsequent buffer exchange which can be problematic for some samples.

Attempts to overcome sample denaturation have included the supplementation of buffers with a variety of detergents (Cheng et al., 2015, Chen et al., 2019), which protect macromolecules from hydrophobic interfaces. However, such approaches require rigorous screening and substantially complicate the optimisation of ice thickness (Glaeser et al., 2016), while many macromolecular complexes are incompatible with suitable detergents. The other popular method to prevent interactions between particles and the air-water interface is to immobilise particles on an appropriate support film. This minimises their recruitment to the air-water interface in the first place, avoiding the issue. An extra amorphous carbon film usually also reduces charge-induced movement, and reduces the concentration of sample required (Grassucci, Taylor, and Frank, 2007), however it significantly increases the background noise, and concomitantly reduces contrast. Several alternative supports, including *titanium silicon* (TiSi) have been suggested (Rhinow and Kühlbrandt, 2008), with graphene (Geim, 2009) currently gaining in popularity due to its extraordinary properties.

Graphene is a single atom thick layer of *sp^2^*-bonded carbon, which is nearly transparent to the electron microscope at the spatial frequencies of interest to cryo-EM (Meyer et al., 2007, Pantelic et al., 2011), thereby introducing minimal background noise. Due to its high conductivity, mechanical strength, single atom thickness and its extremely low noise contribution, which can be removed by Fourier masking, graphene has been proposed as a theoretically ideal support for cryo-EM specimen preparation. The most frequent methodology to isolate single layers of graphene is by *chemical vapor deposition* (CVD) on metal surfaces such as Ni (Reina et al., 2009) and Cu (Li et al., 2009), with Cu supports being more commonly used because this material allows larger graphene growing areas (Batzill, 2012). The usage of Cu-grown graphene monolayers as a support for cryo-EM specimens requires a reliable protocol for transferring the graphene from the metal support to the grid. Pioneering methods achieved the transfer of graphene monolayers using a polymer coating, such as *polymethyl methacrylate* (PMMA) or *polydimethylsiloxane* (PMDS), as a transient support while metal etching was occurring (Reina et al., 2009, Li et al., 2009). However, this technique required wet chemical steps that could easily contaminate and damage the graphene. Currently, the direct transfer of Cu-grown graphene monolayers to cryo-EM grids is well-established (Regan et al., 2010, Naydenova et al., 2019), relying on an initial solvent evaporation step to place the Cu-supported graphene in intimate contact with the grid, and subsequent Cu etching on a FeCl_3_ solution. Using this protocol, graphene-covered grids can be readily obtained, however, the application of sample to these grids remains subject to complications of handling, contamination from the air and destruction of protein samples at the air-water interface, therefore limiting the application of graphene in cryo-EM.

Despite their numerous advantages, graphene monolayers are susceptible to variations in environmental conditions and to hydrophobic airborne contaminants. These accrue within minutes on the graphene surface, increasing their hydrophobicity, and preventing the recruitment of macromolecules. Graphene can be chemically modified to render it hydrophilic, e.g. as graphene oxide (Pantelic et al., 2010, Palovcak et al., 2018), however such methods result in the loss of many of graphene’s desirable properties and increase levels of background noise. Other strategies to render graphene films hydrophilic are to convert graphene to a partially hydrogenated form through hydrogen-plasma cleaning treatment (Russo and Passmore, 2014a) or to gently oxidize graphene surface using ultraviolet (UV) irradiation to generate a small amount of ozone gas to gently oxidize sample surfaces (Han et al., 2020), both of these making the surface more suitable for protein deposition. All of these approaches are expensive and difficult, requiring specialist equipment. Furthermore, these techniques involve air exposure of the graphene films between grid preparation and sample application, thus exposing the graphene films to airborne contaminants, and hence increasing their hydrophobicity (Li et al., 2013).

In this study we have developed methods that bypass the exposure of the surfaces of graphene and carbon to the air entirely, and present a novel support floatation block that enables the direct, wetted, preparation of both amorphous carbon and graphene supported cryo-EM samples, thereby avoiding airborne contamination and thus the requirement for further treatment in each case. The materials required are inexpensive and readily commercially available, sample handling is robust and reproducible, and only small volumes of sample are required (10 µL per well) the majority of which can typically be recovered for re-use if the sample in question is precious. Additionally, the same block allows buffer exchange *in situ* either through injection ports or by direct transfer on mica, for example to quench crosslinked samples, exchange non-vitrifiable buffers, or to perform on-grid affinity purification.

## 2 Results

### 2.1 A support floatation block for simple preparation of cryo-EM samples on carbon and graphene films

**Fig. 1:**
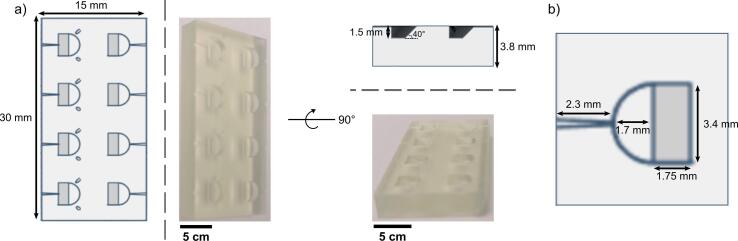
Design of the support floatation block. a) Top and side views of the support floatation block with detailed measurements showing the shape, depth, and ramp of the wells. On the left, a diagram of the block and on the right a real image displaying the same view. b) Inset of a top view representation of one block well with more detailed measurements. The tweezers “groove” can be appreciated from a top view on the left of the well. The volume of each well is just over 10 µL.

We have designed a support floatation block for direct sample preparation on carbon and graphene support films, with multiple applications in different protocols for cryo-EM sample preparation. The block measures 30 mm × 15 mm × 3.8 mm and incorporates two columns of 4 wells evenly distributed on its surface to allow preparation of four samples. This number was chosen as it allows preparation of enough grids to fill a standard grid box (Fig. 1a). Each well accommodates a volume of 10 µL, which is slightly larger than the typical volume of sample used to prepare a cryo-EM grid, however the majority of the sample in the well can be recovered during most applications if required. The wells have a ramp of 40° to facilitate proper release of amorphous carbon from mica sheets during the floatation of carbon films, and to support the mica *in situ* if it is later to be transferred for buffer exchange. One set of wells, in which the ramp approaches the edge of the block, are intended to be used when buffer exchange is being performed, whereas the other set, in which the ramp approaches the centre of the block, are intended to be used when it is not. The width of the wells is 3.4 mm, allowing 0.2 mm clearance on each side over the standard cryo-EM sample grid dimeter (3 mm), to avoid grid contact with the well walls, which could potentially cause damage (Fig. 1b). Each well also has a small groove on the outer side with a small inclination intended to minimise the movement of the tweezers when depositing or lifting grids from the wells, to achieve the same aim (Fig. 1b). The thickness of the block is the same height as the midline of Vitrobot grid-handling tweezers, so that the sample grid will rest parallel to the surface of the well when held and lying within the groove. The wells of one column have small channels on their rounded walls (0.8 mm diameter) which allow buffer exchange *in situ,* using needles and a peristaltic pump. These channels are located at different angles to accommodate two needles at the same time to allow continuous liquid exchange within the well.

## 3 Application of the support floatation block to the preparation of samples on amorphous carbon films

Carbon films, deposited by sputter coating onto mica under vacuum, have been widely used as supports since the dawn of electron microscopy, with several experiments involving the direct transfer of samples to carbon films by floating carbon onto the sample (Valentine et al., 1968, Passmore and Russo, 2016). The support floatation block substantially facilitates the preparation of either negatively stained or cryo-EM grids with an extra layer of amorphous carbon, providing both a container of minimal volume for the sample and the stain solution (10 µL), and an ideal support for the mica while carbon is floating in the liquid. Without an appropriate floatation support, such as the block designed here, carbon films are normally floated in small petri dish, which require a high volume of sample, or in droplets on Parafilm which do not provide an appropriate angle for the carbon to be released from the mica, making it more prone to breakage.

For the application of carbon films, short, single grid-width, strips of carbon coated mica are cut. For cryo-grid preparation, the non-exchange wells are then filled with 10 μL sample. The mica strip is lowered gently into the well, allowing the carbon to be released and to remain on the surface of the sample. Because the carbon film is wetted directly by the sample there is little-to-no air exposure, and no treatment or cleaning of the carbon layer is required in order to allow sample adherence and proper spreading. The block is designed to favour proper carbon release on the 40° ramp located within the wells (Fig. 1a), and to support the mica once it is in place, thus allowing proper floatation of the carbon films on top of liquid. The carbon films are incubated for an appropriate period of time, which varies depending on the sample adherence to carbon (20 s–20 min). Sample populated films are lifted off using a standard carbon coated grid on the sample freezing tweezers, which approach the well from the opposite side from the mica sheet, and then directly blotted and vitrified.

### 3.1 Application of the support floatation block to the preparation of samples on graphene films

**Fig. 2:**
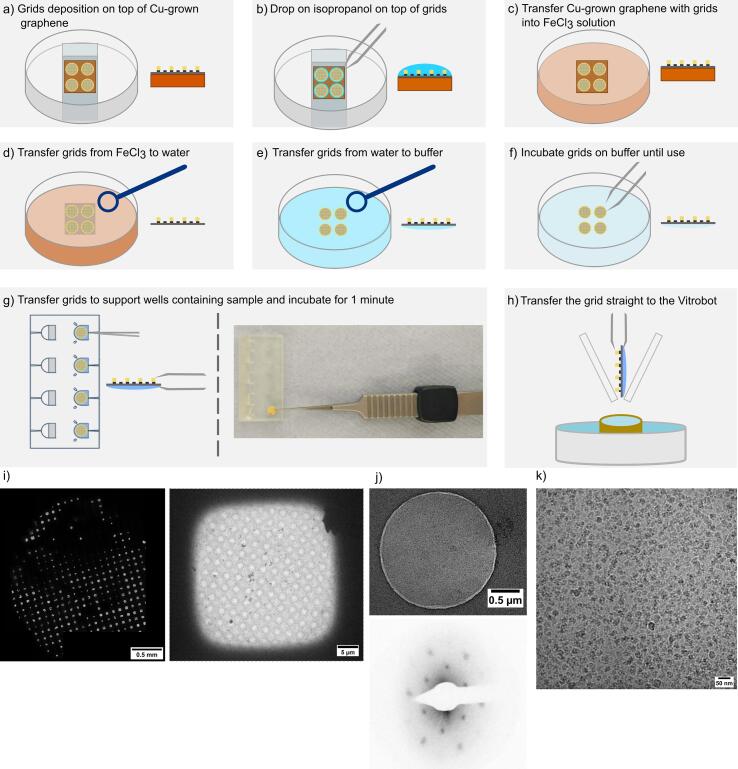
Direct transfer of graphene-covered grids using the support floatation block. a) The Cu-grown graphene sheet is placed onto a glass slide and grids are deposited on top of it, with their carbon side facing downwards. b) A droplet of isopropanol is deposited on top of each grid and left to evaporate. c) The Cu-grown graphene sheet with grids on top is transferred on top of a FeCl_3_ solution and left overnight. d) After proper Cu-etching grids are fished using a loop, e) washed 2 times with water, f) and finally transferred to buffer solution. g) The sample is deposited into the wells of the support floatation block, and grids are picked from the buffer solution and applied to the sample-containing wells. g) After proper incubation, grids can be lifted with the Vitrobot tweezers, with the aid of the tweezer groove at the edge of the support, and h) directly transferred to the Vitrobot for blotting and plunge-freezing into liquid ethane. i) Atlas and grid square view from a graphene-covered grid prepared with the support floatation block. j) Grids were well populated with the sample, and the reciprocal lattice from graphene diffraction was clearly evident. j) Ribosomes were well-preserved, high-contrast, and particle binding was evidently good.

For the use of graphene support films, the process is slightly more prolonged and complicated as such films are typically grown on Cu substrates, rather than being deposited on mica, and must be released with more stringent chemical treatment. Graphene covered grids are transferred from Cu-grown graphene to gold carbon mesh EM grids according to the established direct transfer protocol (Regan et al., 2010) (Fig. 2a–e), involving isopropanol evaporation to produce a tight interaction between the carbon of the EM grid and the graphene layer on the Cu substrate. After graphene adsorption to the grid and etching of the Cu with FeCl_3_, the grids are extensively washed with water and finally transferred with a loop to a glass petri dish containing an identical buffer to that in which the sample has been prepared (Fig. 2f). The grids must be kept wetted at all times to avoid their exposure to airborne contaminants (Li et al., 2013). The sample (10 μL) can then be pipetted into a non-buffer exchange support floatation block well (Fig. 2g). When the sample is ready within the block, a graphene coated grid can be transferred from the buffer container onto the surface of the sample-containing well. After an appropriate incubation period, the grids can be picked with the freezing tweezers and directly blotted and vitrified (Fig. 2h). Throughout this protocol exposure to the air is prevented to minimise airborne contamination of the graphene support film, although this will still accrue over time after Cu-etching so the grids cannot be stored for long periods, and therefore pre-treatment is not required for proper sample spreading. We prepared grids using this methodology with cell-free extracts and imaged them at an F20 FEI microscope operated at 200 keV. Micrographs displayed graphene coverage throughout and appropriate sample spreading, therefore graphene hydrophobicity is not an issue if it is kept wet during the whole sample preparation process (Fig. 2i).

### 3.2 A modified density gradient fixation protocol and application of the support floatation block for subsequent buffer exchange for grids with *in situ* support films

**Fig. 3:**
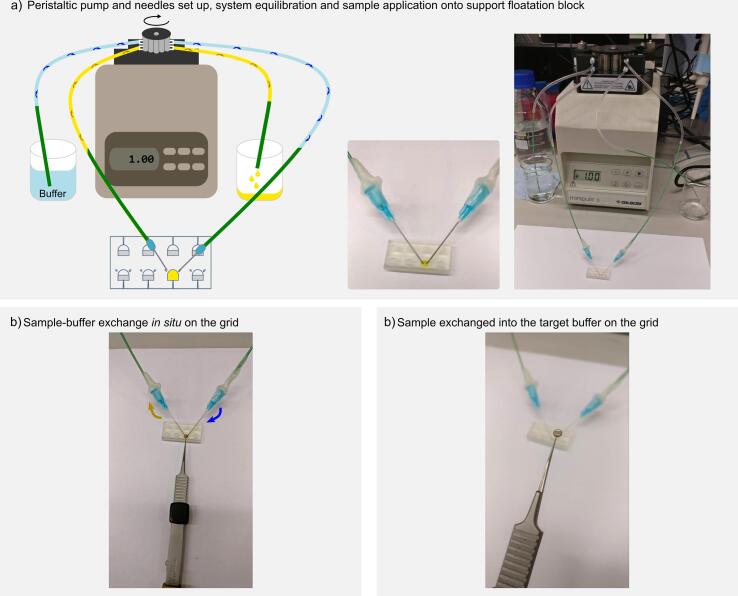
Prefixation protocol and buffer exchange *in situ* with the support floatation block. a) Diagram showing the preparation of PreFix gradients and images of gradients produced in the laboratory. The gradient shown has been tinted using food colouring to visualise the different regions. b) Diagram and images showing the buffer exchange setup using the peristaltic pump and the support floatation block. c) Illustration of buffer exchange on a grid using the support floatation block. The peristaltic pump works in opposition to itself to move the wash buffer through the well at a constant rate. Yellow food colouring has been used to visualise the exchange process, which takes only 10–30 s, even for viscous samples. d) Picture of a grid exchanged into the target buffer. (For interpretation of the references to colour in this figure legend, the reader is referred to the web version of this article.)

**Fig. 4:**
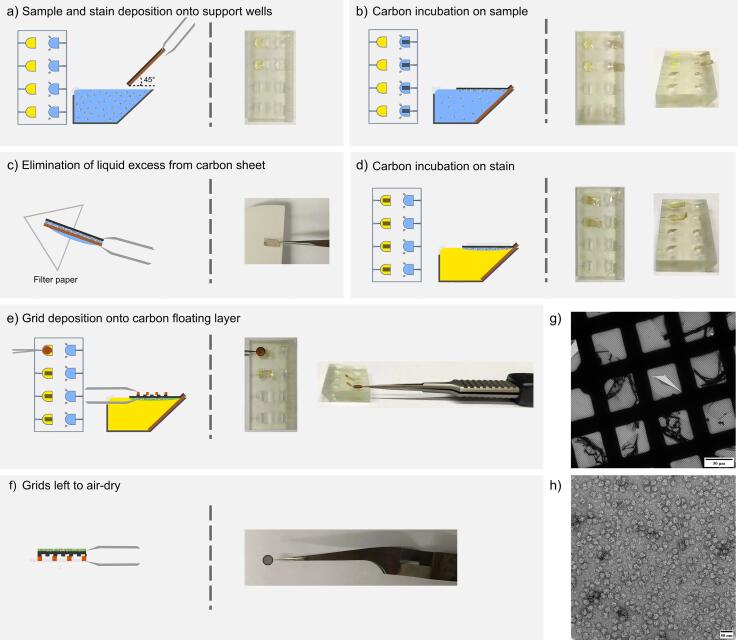
Carbon-floating protocol using the support floatation block to prepare negatively stained samples. a) Sample and stain are deposited onto the wells of the block. b) Carbon films deposited onto mica sheets can be floated onto the sample and left to incubate for 1–5 min. c) Carbon films are recovered by picking the mica sheets with tweezers and excess liquid removed by blotting the mica over a filter paper. d) Carbon films are transferred to stain (2.0% (w/v) uranyl acetate in water in this case) and left to incubate for one minute. e) Finally, the carbon films can be picked using the carbon-coated side of a grid by touching the floating carbon layer on the stain, with the aid of the tweezers *groove* of the block. f) The grid is left to air-dry with the carbon side facing upwards. g) Low magnification image of a grid prepared using this protocol. A slightly broken section has been chosen to allow folds revealing the carbon layer to be clearly observed. h) A representative micrograph from a grid prepared with this protocol using a cell free extract as sample.

Sample heterogeneity originating from sample instability is an important issue when purifying dynamic macromolecular complexes. Unfortunately, the most common protocols to address these problems involve the introduction of buffer components incompatible with vitrification. Kastner and colleagues established a protocol based on the combination of centrifugation and mild fixation, “GraFix” (Kastner et al., 2008, Stark, 2010), which successfully stabilises protein complexes while diluting weak aggregates and other contaminants. In our laboratory, we more typically adopt a variation of this approach, a pre-fixation gradient protocol incorporating quenching, “PreFix”, which was successfully used for the preparation of the mTORC2 complex (Stuttfeld et al., 2018). Instead of adding the fixation agent to the denser buffer, an extra layer of the less dense buffer supplemented with cross-linking agent is added on the top of the upper buffer and the denser buffer is supplemented with a quenching solution (Fig. 3a). The sample is deposited on top of the gradient and is subjected to ultracentrifugation, thereby being both fixed and quenched *in situ*. This minimises dissociation, which can occur during centrifugal separation, while providing a quenched, and therefore stable, final sample. An illustration of the application of this protocol to MS2 virions (de Martín Garrido et al., 2019) is shown in Fig. 4a. In all cases in which gradient purification is performed, the sample buffer after recovery of the band is incompatible with vitrification, containing cryo-protectant solutions.

We have designed our support floatation block to allow buffer exchange for such gradient derived samples with a simple buffer exchange methodology. Two needle ports are present within the print to allow continuous flow through the 10 μL well. An inexpensive two-tube peristaltic pump is used to pump buffer both into and out of the well at the same rate. This set-up must be pre-equilibrated into the target buffer before the sample is applied to the buffer exchange well. There is typically some small variation in the surface level of the well as the pump pressure oscillates, however this is not sufficient to damage the grid or support film. In the case of samples derived from gradients, because the vitrification buffer is substantially less dense than the gradient buffer encompassing the sample, there is little mixing and a layer of vitrifiable buffer quickly forms under the grid, allowing exchange to occur quite quickly, typically within seconds. An illustration of this process is shown in Fig. 3(b–d).

### 3.3 Application of the support floatation block to buffer exchange for carbon support films on mica

The support floatation block is also designed to allow buffer exchange for such gradient derived samples by direct movement. For carbon films on mica, the sample is pipetted into the buffer exchange well, and the buffer to be exchanged into (i.e 2% Uranyl Acetate for negative staining) is applied to the opposing non-buffer exchange well. After the initial incubation on sample, the carbon film is recovered by withdrawing the mica sheet very slowly to minimise residual viscous sample retention, blotted carefully with filter paper to remove excess liquid, and subsequently buffer exchanged by application to the opposing well (Fig. 4c, d). The floating carbon layer is then recovered with the carbon-covered side of a washed and plasma cleaned EM grid (Fig. 4e). After incubation, the grid can be lifted and immediately blotted and vitrified. Negatively stained EM grids can also be prepared according to an essentially identical methodology, adapted from Valentine’s floating carbon technique (Valentine, Shapiro, and Stadtman, 1968), which is illustrated using a cell-free reaction as sample (Fig. 4f). Micrographs show carbon coverage throughout the grid (Fig. 3g) as well as homogeneous sample distribution around the carbon. Additionally, particles are sufficiently stained, without low contrast being an issue (Fig. 3h). The use of the block dramatically improves the ease of grid handling during the carbon-floatation technique, and minimises the exposure to air of both the sample and the carbon support film grids.

## 4 Discussion

In this study we report improved protocols for the handling of both amorphous carbon and graphene films in cryo-EM sample preparation. Our approaches involve the use of a sample floatation block that we have specifically designed for these purposes and made freely available to be 3D printed or ordered from any 3D printing company with a suitable SLA printer.

The use of a support floatation block and direct transfer of carbon and graphene films to the sample avoids the requirement for plasma cleaning or other treatment, which is particularly advantageous when working with graphene as these requirements are particularly onerous in this case. We note that both carbon deposited on mica sheets and graphene monolayers on copper sheets, as well as SLA prints and FeCl_3_, are commercially available at low cost (hundreds of USD), however the equipment required to render graphene hydrophilic without destroying it (hydrogen plasma cleaners, high-power UV apparatus, or ozone generators) is expensive (tens of thousands of USD), specialist, and prohibitive for many laboratories. Because contamination accrues from the air, graphene grids cannot be purchased and used without treatment. Alternatively, graphene can be converted to graphene oxide (Pantelic et al., 2010, Palovcak et al., 2018), however it usually introduces a high background signal and is less effective in neutralising accumulated surface contamination (Gómez-Navarro et al., 2007), which makes it a less ideal substrate for EM than graphene.

Our approaches using the novel support floatation block minimise sample contact with the air-water interface, thereby avoiding both sample denaturation and support film contamination. Furthermore, we have optimised the design of the block for small sample volumes, to make it as applicable as possible to any and all single particle EM purposes, from negative stain to high-resolution cryo-EM, and to allow on-grid buffer exchange either by transfer of the film between wells, or *in situ*. The equipment required for this is minimal (tweezers, needles and a peristaltic pump), and both set-up and operation are quick, simple, and robust. In most cases the majority of the sample can also be recovered, which is both important and beneficial when working with precious low-yield samples. The only notable disadvantage of which we are aware is that on some vitrification equipment the grid must be loaded, with the sample *in situ*, close to the surface of the liquid ethane cryogen, which is undesirable, but this does not prevent the practical adoption of these approaches. Additionally, we also present a modification of the GRAFIX protocol (Kastner et al., 2008, Stark, 2010) that allows prefixation and quenching of multiprotein complexes *in situ*, which proves to be very advantageous for the preparation of labile and dynamic complexes (Stuttfeld et al., 2018).

We believe that the protocols reported here are likely to be of utility to many single particle cryo-EM investigations, as support films are required for many single particle projects. Furthermore, we are optimistic that the advent of affinity grids (Kelly et al., 2010, Wang et al., 2020) will substantially increase the utility of our apparatus to the field in the near future as the field moves towards on-grid affinity purification from small sample volumes, e.g. from a cell-free protein expression, which will necessarily entail a requirement for controlled *in situ* buffer exchange on grids.

## 5 Materials and methods

### 5.1 Preparation of exemplar samples

To demonstrate both the direct transfer of graphene-covered grids and carbon-floating negative staining, cell-free extract reactions from the PURExpress *In Vitro* Protein Synthesis Kit (NEB, #E6800S) were used. A standard reaction mix contains 10 μL Solution A and 7.5 μL Solution B and nuclease-free water to 25 μL of final volume. The reactions were scaled up and down accordingly. To demonstrate the prefixation gradient, we used an MS2 virion provided by Michael Crone (de Martín Garrido et al., 2019).

### 5.2 Pre-fixation density gradient preparation

We prepared 10–40% glycerol (v/v) gradients from 20 mM HEPES pH = 7.6, 10 mM MgCl_2_, 30 mM KCl and 20 mM Tris pH = 8.0, 10 mM MgCl_2_ and 30 mM KCl, respectively. Half the solution of the lower buffer (40% glycerol-Tris) was supplemented with 200 μL of red food colouring (Waitrose, London, United Kingdom) for the purposes of visualisation. We supplemented 5 mL of the upper buffer with 0.025% glutaraldehyde (10% glycerol-HEPEs) and green food colouring (Waitrose, London, United Kingdom) for the purposes of visualisation. After gradient formation, some liquid was removed from the top, to add a layer of glutaraldehyde solution and, on top of that, a further layer of top buffer. Gradients were centrifuged at 30 000 rpm, 4 °C for 16 h. The sample was visualised through light scattering using a Piston Gradient Fractionator (Biocomp, Fredrickton, New Brunswick, Canada).

### 5.3 Design and production of the support floatation block

The support floatation block was designed using Tinker CAD software (AutoDesk, California, United States of America). The STL file has been deposited and is freely available from the public Thingiverse repository [www.thingiverse.com/thing:3440684] (MakerBot, New York, United States of America), or on request. Support floatation blocks were produced using a Formlabs Form 3 SLA 3D printer at the Imperial College Advanced Hackspace. The plastic used to print the block must be water resistant, smooth and poorly surface active to minimise sample loss during preparation. Dental resin is a highly suitable, widely available, material (Formlabs dental resin, Formlabs, Somerville, MA, USA). We printed the blocks shown here using transparent resin for ease of demonstration.

### 5.4 Carbon film preparation

Mica sheets (Agar Scientific, Stansted, United Kingdom) were cleaved to yield an ultra-flat surface and placed in a Quorum Q150T coating system (Quorum Technologies, Lewes, United Kingdom) equipped with a film thickness monitor. They were coated from 3.05 mm carbon rods, from an initial vacuum of 5 × 10^−5^ mbar, using automatic ramping to prevent sparking and with a target film thickness of 2 nm.

### 5.5 Graphene film preparation

After washing with distilled water and ethylacetate, Quantifoil R 2/1, 300 Mesh Gold grids were covered with graphene using an established protocol for the direct transfer from Cu-Grown graphene to cryo-EM grids (Regan et al., 2010). Washed grids were placed on top of a Cu-grown graphene sheet (Graphenea) deposited onto a glass slide, and each grid was covered with a drop of isopropanol, thus allowing intimate contact between the monolayer graphene and the grid. When the isopropanol was completely evaporated, the Cu-grown graphene sheet with grids on top was floated onto a 10% (w/v) FeCl_3_ solution contained on a glass Petri dish and left to etch at room temperature. After Cu-etching, grids floating onto a graphene monolayer (visible by eye with suitable lighting) were fished with a loop and transferred to distilled water. Grids were washed two times in water to remove all iron chloride and finally transferred into a Petri dish containing buffer until sample preparation.

### 5.6 Graphene cryo-grid preparation and data collection

An aliquot of PURExpress reaction (25 µL) was diluted to a final volume of 60 µL and deposited to the block wells (10 µL each). Graphene-covered grids were transferred from buffer to the block wells containing the sample and left floating on sample for 1 min. After proper incubation, grids were picked with tweezers, with the aid of the groove located on the sides of the block, and transferred to the Vitrobot Mark IV (FEI) chamber at 4 °C and >95% humidity, where they were subsequently blotted for 2.5 s and plunge-frozen in liquid ethane. Frozen grids were imaged on a Tecnai F20 (FEI) operated at 200 kV, using an early FEI Falcon II integrating detector, at a nominal magnification of x81k, with a nominal dose of ~50 e/Å^2^ and pixel size 1.4 Å/pixel.

### 5.7 Negative stain buffer exchange on mica

Quantifoil R1.2/1.3 300 mesh copper grids were prepared by washing with double distilled water and ethylacetate, before plasma cleaning for a duration of 10 s. The reaction products from the PURE system were applied to amorphous carbon using the floating method shown (Fig. 3) and subsequently exchanged to 2% (w/v) uranyl acetate, with the aid of the support floatation block. The carbon floating on the stain was then transferred onto plasma-cleaned grids, which were left to air-dry. Grids were imaged on a Tecnai T12 Spirit (FEI) operated at 120 kV, at a nominal magnification of 67kx and acquired over an applied defocus range of −1 μm to −1.5 μm.

### 5.8 PreFix buffer exchange *in situ*

A peristaltic pump (Gilson, Wisconsin, United States of America) with two identical sets of tubing was connected such that one was pumping out through a needle, while the other was pumping in through another. The needles were inserted into a buffer exchange well within a support floatation block, and the system then equilibrated into the desired sample buffer for exchange at a minimal rate of flow. Once equilibrated, the peristaltic pump was stopped, the well emptied of buffer and 10 µL of sample pipetted into the well. The film-coated grid was then applied to the sample and incubated for one minute. Finally, the peristaltic pump was run at the same flow rate and direction. When the apparatus was tested with a pre-fixation gradient sample doped with food colouring in order to establish its effectiveness, complete exchange of the buffer was accomplished within thirty seconds (Fig. 3b–d).

## CRediT authorship contribution statement

**Natàlia de Martín Garrido:** Conceptualization, Methodology, Validation, Investigation, Writing - original draft, Writing - review & editing, Visualization. **Wencheng Fu:** Methodology, Writing - original draft. **Kailash Ramlaul:** Validation, Writing - review & editing. **Zining Zhu:** Methodology. **David Miller:** Methodology, Resources. **Daniel Boehringer:** Conceptualization, Writing - review & editing. **Christopher H.S. Aylett:** Conceptualization, Methodology, Writing - review & editing, Supervision, Project administration, Funding acquisition.

## Declaration of Competing Interest

The authors declare that they have no known competing financial interests or personal relationships that could have appeared to influence the work reported in this paper.
