# Corrigendum to “Direct transfer of electron microscopy samples to wetted carbon and graphene films via a support floatation block” [J. Struct. Biol. 213 (2021) 107677]

**DOI:** 10.1016/j.jsb.2021.107739

**Published:** 2021-09

**Authors:** Natàlia de Martín Garrido, Wencheng Fu, Kailash Ramlaul, Zining Zhu, David Miller, Daniel Boehringer, Christopher H.S. Aylett

**Affiliations:** a Section for Structural and Synthetic Biology, Department of Infectious Disease, Imperial College London, London, United Kingdom; b State Key Laboratory of Microbial Metabolism, School of Life Sciences & Biotechnology, Shanghai Jiao Tong University, Shanghai, China; c Imperial College Advanced Hackspace, Imperial College London, London, United Kingdom; d Cryo-EM Knowledge Hub (CEMK), ETH Zurich, Zurich, Switzerland

The authors regret that Fig. 3 was truncated in the original publication. Here we provide the complete original figure as submitted to the journal during peer-review of the manuscript.

**Figure:**
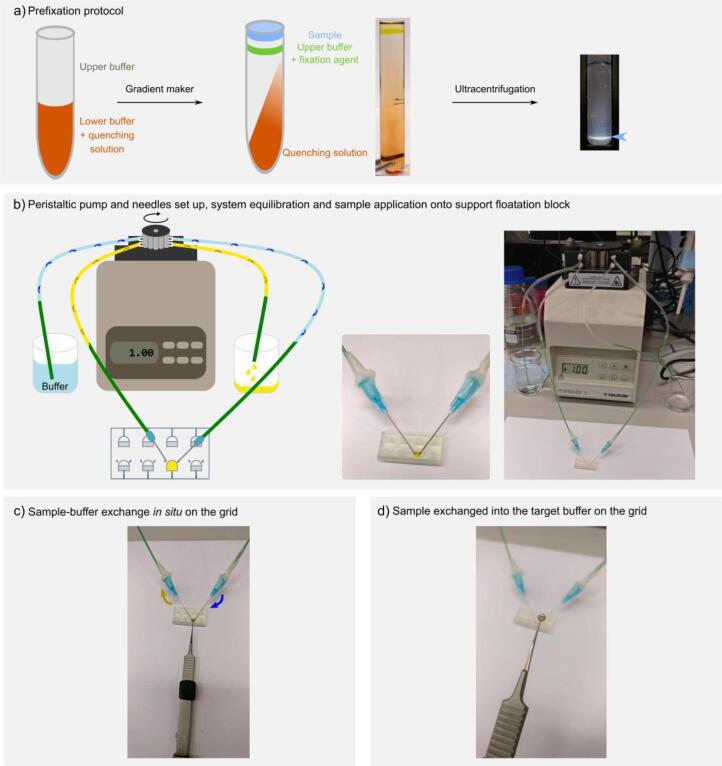

The authors would like to apologise for any inconvenience caused.

